# Complex of C_60_ Fullerene with Doxorubicin as a Promising Agent in Antitumor Therapy

**DOI:** 10.1186/s11671-015-1206-7

**Published:** 2015-12-29

**Authors:** Svitlana V. Prylutska, Larysa M. Skivka, Gennadiy V. Didenko, Yuriy I. Prylutskyy, Maxim P. Evstigneev, Grygoriy P. Potebnya, Rostyslav R. Panchuk, Rostyslav S. Stoika, Uwe Ritter, Peter Scharff

**Affiliations:** Taras Shevchenko National University of Kyiv, 64 Volodymyrska Str., 01601 Kyiv, Ukraine; R.E. Kavetsky Institute of Experimental Pathology, Oncology and Radiobiology of NASU, Vasylkivska Str. 45, 03022 Kyiv, Ukraine; Belgorod State University, Pobedy Str.85, 308015 Belgorod, Russia; Institute of Cell Biology, NAS of Ukraine, 14-16 Drahomanov Str., 79005 Lviv, Ukraine; Technical University of Ilmenau, Institute of Chemistry and Biotechnology, 25 Weimarer Str., 98693 Ilmenau, Germany

**Keywords:** C_60_ fullerene, Doxorubicin, Antitumor effect, Antimetastatic effect, Immune response

## Abstract

The main aim of this work was to evaluate the effect of doxorubicin in complex with C_60_ fullerene (C_60_ + Dox) on the growth and metastasis of Lewis lung carcinoma in mice and to perform a primary screening of the potential mechanisms of C_60_ + Dox complex action. We found that volume of tumor from mice treated with the C_60_ + Dox complex was 1.4 times less than that in *control* untreated animals. The number of metastatic foci in lungs of animals treated with C_60_ + Dox complex was two times less than that in *control* untreated animals. Western blot analysis of tumor lysates revealed a significant decrease in the level of heat-shock protein 70 in animals treated with C_60_ + Dox complex. Moreover, the treatment of tumor-bearing mice was accompanied by the increase of cytotoxic activity of immune cells. Thus, the potential mechanisms of antitumor effect of C_60_ + Dox complex include both its direct action on tumor cells by inducing cell death and increasing of stress sensitivity and an immunomodulating effect. The obtained results provide a scientific basis for further application of C_60_ + Dox nanocomplexes as treatment agents in cancer chemotherapy.

## Background

Suppression of proliferative activity of tumor cells is a basic strategy when using traditional chemotherapeutic drugs [1]. Doxorubicin (Dox) is the anthracycline antibiotic widely used for treatment of cancers of different origin [2]. It uses two main mechanisms in cytotoxic action towards tumor cells: intercalation into DNA helixes followed by inhibition of the DNA synthesis and generation of free radicals followed by DNA impairment and cell membrane damage [3]. However, the antitumor effect of traditional chemotherapy is always associated with numerous negative side effects, in particular, the toxicity towards cells of normal organs and tissues. Dox causes potent oxidative stress, mitochondrial dysfunction, and Bcl-2 expression disturbance followed by the apoptotic damage in heart tissue. A number of substances with ability to attenuate the Dox-induced cardiotoxicity have been developed nowadays in order to improve the outcome of the long-term treatment with Dox [4]. In that regard, C_60_ fullerene is a promising carbon nanostructure that is characterized by unique physical and chemical properties [5] and biological activity both in vitro and in vivo [6].

It was established that pristine C_60_ fullerenes at low concentrations are nontoxic [7, 8] and they are able to penetrate through the cytoplasmic membrane of treated cells [9]. One of the biologically most relevant features of C_60_ fullerene is its antioxidant effect [10]. Our previous results also revealed antioxidant properties of pristine C_60_ fullerene [11].

C_60_ fullerene and its derivatives possess potent anticancer activity [12]. It has been reported that C_60_ fullerene nanocrystal induces certain hallmarks of autophagy in cancer cells [13]. The tumor inhibitory effect of fullerenes is accompanied by the immunomodulatory activity [14].

It is important to emphasize that chemical modification of the surface of C_60_ molecule for improvement of its water solubility often leads to changes in its physical and chemical properties and to a decrease in specific biological effects. Thus, utilization of pristine C_60_ fullerene would be more desirable. In our previous study [15], we showed that the water-soluble pristine C_60_ fullerene directly suppresses growth of transplanted malignant tumor. Chen et al. [16] reported that antitumor effect of pristine and functionalized C_60_ fullerene might be associated with modulation of the oxidative stress and the anti-angiogenic and immunostimulatory activity. Injac et al. [17] demonstrated the ability of fullerenol to decrease the acute Dox pulmotoxicity in rats with malignant neoplasm through inhibition of oxidative stress. In our previous study [18], it was shown that the combination of Dox with C_60_ fullerene resulted in increase of therapeutic efficacy of the treatment. Taking into account these data, we also suggested [19, 20] that Dox immobilization on C_60_ fullerene (C_60_ + Dox complex formation) can reduce negative side effects of this drug towards normal cells as well as enhance its ability to enter target tumor cells.

The main goal of this work was to (1) evaluate the effect of C_60_ + Dox complex on growth and metastasis of Lewis lung carcinoma (LLC) and (2) perform primary screening of the potential mechanisms of C_60_ + Dox action.

## Methods

### Material Preparation and Characterization

The highly stable aqueous colloid solution of purified C_60_ fullerene (C_60_FAS; concentration 0.15 mg/ml) was prepared as reviewed in [21, 22]. The method is based on the technology of transferring C_60_ molecules from toluene to an aqueous phase with the help of ultrasonic treatment.

The atomic force microscopy (AFM) data [21–23] confirm randomly arranged individual C_60_ molecules with a diameter of ~0.7 nm and their bulk sphere-like aggregates with a height of 2–100 nm in C_60_FAS.

Dox (“Doxorubicin-TEVA”, Pharmachemie B.V.) was dissolved in saline at initial concentration 0.15 mg/ml and used in all experiments. It was immobilized on the С_60_ fullerene according to the following protocol: C_60_FAS (0.15 mg/ml) and Dox (0.15 mg/ml) were mixed in 1:2 volume ratio, and the resulting mixture was treated for 20 min in the ultrasonic disperser. After that, it was subjected to 12-h magnetic stirring at room temperature. Pronounced hypochromic effect observed in spectrophotometric experiment and AFM data clearly indicate a formation of stable C_60_ + Dox complex [19, 20].

### Animals

The male С57Bl/6 mice (20–21 g weight) were kept at 298 ± 1 K on a standard diet in the vivarium of R.E. Kavetsky Institute of Experimental Pathology, Oncology and Radiobiology, NAS of Ukraine (Kyiv). All experiments were conducted in accordance with the international principles of the European Convention for protection of vertebrate animals under the control of Bio-Ethics Committee of that institution.

### Tumor Model, Treatment Regimens, and Study Design

LLC was used as an experimental model. LLC cell line was obtained from the cell line bank of the R.E. Kavetsky Institute of Experimental Pathology, Oncology and Radiobiology, NAS of Ukraine (Kyiv). Tumor cells (5 × 10^5^ in the volume of 100 μl) were transplanted intramuscularly into the mouse limb. After transplantation of tumor cells, the experimental animals were randomized by weight and distributed in four groups with ten animals per group:

- *Group 1* (C_60_ fullerene injection). C_60_FAS was used in 1.5 mg/kg dose (0.2 ml) injected intraperitoneally to mice with transplanted tumor once per day for 5 days with a day interval [15].
- *Group 2* (Dox injection). Dox was used in 1.5-mg/kg dose (0.2 ml) injected intraperitoneally to mice with transplanted tumor once per day for 5 days with a day interval [24].
- *Group 3* (C_60_ + Dox complex injection). C_60_ + Dox mixture was used in 1.5-mg/kg dose (0.2 ml) injected intraperitoneally to mice with transplanted tumor once per day for 5 days with a day interval.
- *Control*. The mice with transplanted tumor were injected with saline (0.2 ml) once per day for 5 days with a day interval.

*Intact animals* were used in order to investigate immunological indices (cytotoxic activity of the peritoneal macrophages and mononuclear splenic leucocytes).

The injections of C_60_ fullerene, Dox, or C_60_ + Dox complex were started on the 2nd day after tumor cell transplantation. The protocol of injecting C_60_ fullerenes was based on the fact that C_60_ fullerenes administered intraperitoneally to rats (500 mg/kg) were subjected to clearance from the organism within 2–4 days [25]. The C_60_ fullerene dose applied in our experiments was significantly lower than the LD_50_ value determined for C_60_ fullerene which, after oral administration to mice, was equivalent to 600 mg/kg of body weight [25].

The kinetics of tumor growth was evaluated as described [15] by linear dimensions of tumor measured every third day with the use of calipers starting from the 9th day after tumor cell inoculation. The euthanasia of experimental animals was performed at the end of the experiment (22nd day), and the number and size of metastases in animal lungs were monitored.

Anticancer effect was also characterized by growth inhibition index, GII, calculated by the formula GII = (*V*_c_ ‐ *V*_exp_)/*V*_c_ × 100 %, where *V*_c_ and *V*_exp_ are the average volumes of tumor of control and experimental animals, respectively; $V=1/2\cdot {\left(\frac{a+b}{2}\right)}^3$, where *а* and *b* are the length and width (in millimeters) of the tumor site, respectively [15].

### MTT Assay

To analyze cytotoxic activity of the peritoneal macrophages and mononuclear splenic leukocytes, the modified MTT assay was used as described [26]. Cytotoxic activity of the studied samples was calculated using the formula Cytotoxicity index = (1–ε/ε_c_) × 100 %, where ε_c_ and ε are the extinctions of control and test sample, respectively. Measurement of extinction was performed on a digital spectrophotometer (μQuant, BioTEK, USA) at the wavelength of 540 nm.

The investigation of cytotoxic activity of immunocytes was performed on the 22nd day after tumor cell transplantation. Suspension of tumor cells was prepared from tissue homogenates. Mononuclear splenic leukocytes were obtained from splenocyte suspension by centrifugation (1500 rpm, 40 min) in Ficoll-Hypaque density gradient (*p* = 1.077). Peritoneal macrophages were isolated without preliminary stimulation. Mice were sacrificed, and peritoneal macrophages were harvested using phosphate-buffered saline containing 100 U/ml of heparin. Cells were centrifuged at 300×*g* for 5 min at 4 °C, washed twice with serum-free DMEM, and re-suspended in DMEM containing 10 % FCS and 40 μg/ml gentamicin.

To perform cytotoxic assay, LLC cells were placed in 96-well plates (3 × 10^5^ cells/well), and mononuclear splenic leukocytes or peritoneal macrophages were added at 20:1 ratio. Cells were incubated in a RPMI-1640 medium supplemented with gentamicin sulfate (100 μg/ml) and maintained at 37 °C for 18 h in 5 % CO_2_ atmosphere. After incubation, MTT (Sigma) was added to a final concentration of 0.5 mg/ml followed by culturing for 3 h. After culturing, cells were centrifuged at 4000 rpm (1600×*g*) for 10 min. Culture medium was removed, and blue formazan crystals were dissolved in 100 μl DMSO. Optical density was determined at 570 nm.

### Western Blot Analysis

The tumors were surgically removed, and cell lysates were prepared by EDTA extraction. After removal of unlysed cell remnants and nuclei by centrifugation in the Eppendorf micro-centrifuge (5 min, 10,000 rpm, 10,200×*g*); protein concentration was determined by standard method, as described [27]; and 10 μg of equal amounts of protein was loaded into 15 % polyacrylamide gel. Proteins were resolved and transferred to Immobilon-P membrane (Millipore, Billerica, MA) using semi-dry transfer (Bio-Rad, Hercules, CA). After incubating the membrane in the blocking buffer, the membrane was incubated with heat-shock protein 70 (HSP70) monoclonal antibodies (Santa Cruz Biotechnology, Santa Cruz, CA). For a loading control, the levels of expression of the β-actin were detected in each sample using mouse β-actin monoclonal antibodies (Sigma). Immunoreactive bands were visualized by chemiluminescence using horseradish peroxidase-conjugated IgG antibodies and ECL Kit (Amersham, Uppsala, Sweden) according to the instructions of the manufacturer.

### Statistical Analysis

For statistical analysis of the obtained results, standard variation data within a group was calculated together with a statistical reliability of differences between two groups of data assessed by Student’s *t* test. The level of significance was set to *p* < 0.05.

## Results and Discussion

### Treatment with C_60_ + Dox Complex Results in the Inhibition of Tumor Growth and Metastasis and Increases Stress Sensitivity of Tumor Cells In Vivo

Experimental animals tolerated the treatment well and exhibited normal behavior, as determined by the activity level and grooming behavior throughout the study.

**Fig. 1:**
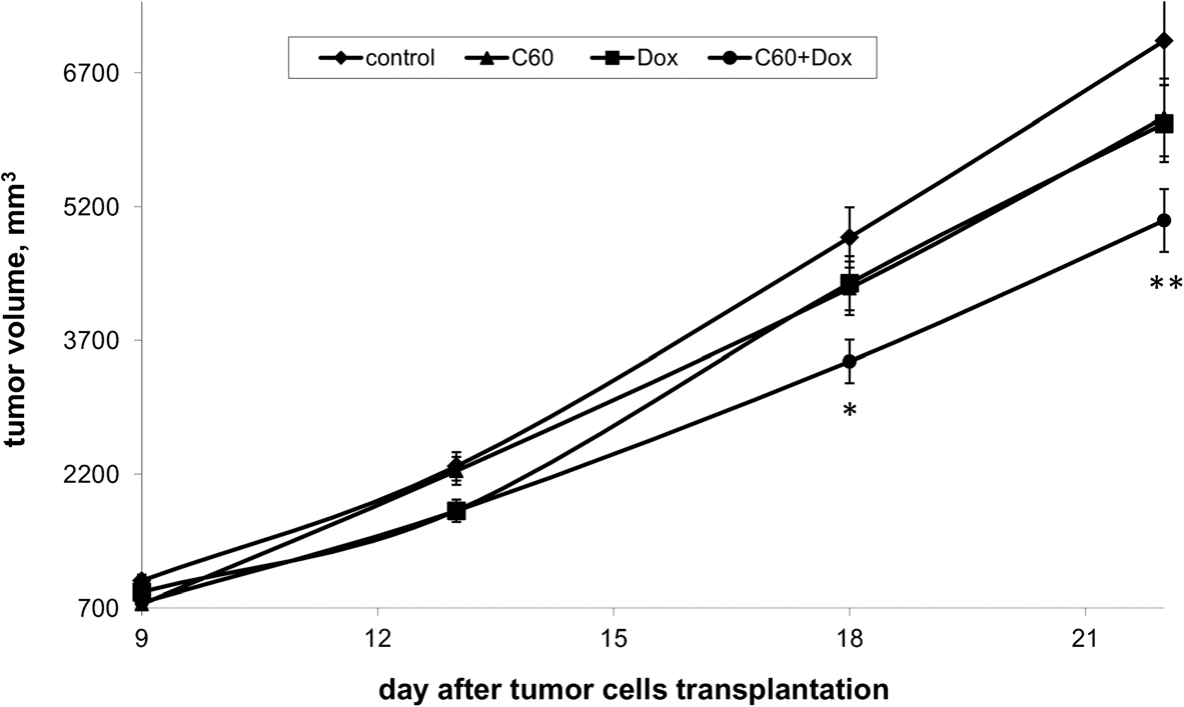
The effect of treatment with C_60_ fullerene, Dox, and C_60_ + Dox complex on tumor volume in LLC-bearing mice; the differences are statistically valid compared to the *control* (*t* test; *p < 0.05, **p < 0.01)

LLC was characterized by a significant growth of its size (volume) from the 9th to the 22nd day of the experiment (Fig. 1). One can see that all applied treatments (C_60_ fullerene, Dox, and C_60_ + Dox complex) caused a decrease (comparing to *control*, i.e., untreated mice) of tumor volume. Tumor volume in animals of *group 1* (C_60_ fullerene injection) and *group 2* (Dox injection) differed slightly. The volume of tumor from mice treated with the C_60_ + Dox complex was significantly lower than that in *control* untreated animals, viz. by 1.4 times.

The effect of C_60_ fullerene and Dox used alone and in C_60_ + Dox complex on tumor growth was evaluated by the GII value presented in Table 1.

**Table 1:** Tumor growth inhibition index (GII, %) for each experimental group on the 9th, 13th, 18th, and 22nd day after tumor inoculation

Animal group	Day after tumor cell transplantation			
	9	13	18	22
Group 1 (C_60_ fullerene injection), *n* = 10	26	20	22	12
Group 2 (Dox injection), *n* = 10	13	22	21	13
Group 3 (C_60_ + Dox injection), *n* = 10	24	22	29	29

Thus, on the 13th day after cancer cell inoculation, tumor volume in mice treated with Dox and C_60_ + Dox complex was 22 % less than that in the untreated animals. There was no inhibition of tumor growth in mice treated with C_60_ fullerene alone. On the 18th day after cancer cell transplantation, we observed the most expressed retardation of tumor growth in animals treated with C_60_ + Dox complex, and the GII value in mice of that group was 29 %. At that time point, the tumor volume in mice treated with C_60_ fullerene and Dox was ~22 % less than that in the untreated animals. On the 22nd day after cancer cell inoculation, the GII value in mice treated with C_60_ fullerene and Dox was decreased by ~13 %, but it did not change in mice treated with C_60_ + Dox complex.

The treatment of tumor-bearing mice with C_60_ fullerene, Dox, and C_60_ + Dox complex caused an inhibition of metastasis of the experimental tumor (Table 2). The number of the metastatic foci in lungs of animals of the group treated with C_60_ + Dox complex was two times less than that in the untreated animals and 1.4 times less than that in mice treated with Dox alone. It should be noted that the metastatic foci in mice treated with C_60_ fullerene, Dox, and its complex were characterized by different sizes. While in the *control* group, large metastatic foci that infiltrated into the lung parenchyma were observed; the metastatic foci were much smaller and solitary in mice treated with Dox and C_60_ + Dox complex. In mice treated with C_60_ + Dox complex, the metastatic foci with the diameter of ≥3 mm were absent. Since only tumor growth beyond the size of 1–2 mm is angiogenesis-dependent [28], we suggested that the small-sized metastatic focus (≤1 mm in diameter) is in a state of dormancy. Therefore, one can suppose that C_60_ + Dox complex exerts a negative effect towards tumor angiogenesis.

**Table 2:** The effect of C_60_ fullerene and Dox used alone and in C_60_ + Dox complex on the LLC metastases

Animal group	The number of tumor nodules in lungs							
	Nodule diameter (mm)							Total number
	<0.5	0.5	1	2	3	4	5	
Control, *n* = 10	12	9	33	25	3	7	11	100
Group 1 (C_60_ fullerene injection), *n* = 10	20	22	23	8	9	1	1	84
Group 2 (Dox injection), *n* = 10	14	5	25	16	7	1	1	69
Group 3 (C_60_ + Dox injection), *n* = 10	16	10	6	16	–	–	–	48

**Fig. 2:**
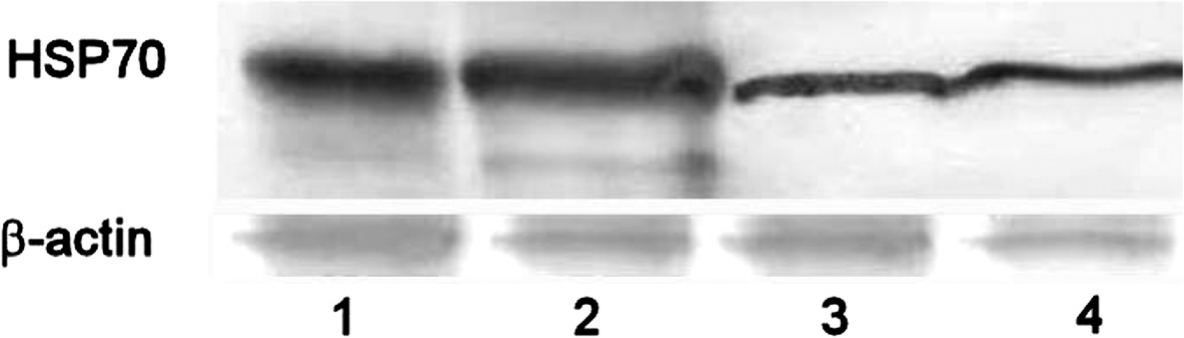
The effect of treatment with C_60_ + Dox complex on the level of HSP70 in tumor tissue of animals with LLC (representative Western blot). *1* and *2*—control animals (untreated); *3* and *4*—C_60_ + Dox-treated animals

It is known that HSP70 is aberrantly expressed in cancer cells of different origins. The survival of these cells strongly depends upon HSPs due to their role not only in protein refolding and degradation but also in preventing apoptosis [29]. Elevated expression of HSP70 is associated with poor response of tumor cells to chemotherapy, and inhibition of its expression was shown to be an effective strategy against cancer [30]. Therefore, we have measured the level of HSP70 in tumor tissue of animals treated with Dox and C_60_ + Dox complex. Western blot analysis of tumor cell lysates revealed a significant decrease in HSP70 level only in animals treated with C_60_ + Dox complex (Fig. 2). The level of HSP70 in tumor lysates from mice treated with Dox alone did not differ from that in the untreated tumor-bearing mice.

The obtained results clearly demonstrate that the anticancer activity of Dox is not only well preserved in its complex with C_60_ fullerene, but it is even enhanced after formation of such complex.

### C_60_ + Dox Complex Modulates Immunological Reactivity of Tumor-Bearing Mice

С_60_ fullerene and its derivatives were shown to possess the immunomodulating properties [14]. Thus, we supposed that the immunomodulating effect of С_60_ + Dox complex can be involved in its antitumor action. To testify this hypothesis, the cytotoxic activity of mononuclear splenic leukocytes and macrophages towards autologic tumor cells was evaluated in tumor-bearing mice treated with the С_60_ + Dox complex.

**Fig. 3:**
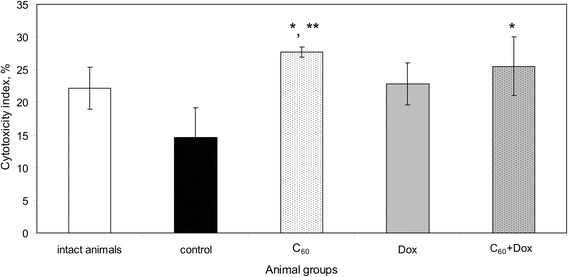
The effect of treatment with C_60_ fullerene, Dox, and C_60_ + Dox complex on the cytotoxic activity of peritoneal macrophages towards autologic tumor cells in LLC-bearing mice; *the differences are statistically valid compared to the *control* (*t* test; *p* < 0.05); **the differences are statistically valid compared to the *intact animals* (*t* test; *p* < 0.05)

Growth of experimental tumor was associated with a decrease of macrophage cytotoxicity towards autologic tumor cells in vitro (Fig. 3).

Treatment with Dox as well as with С_60_ fullerene used alone and in С_60_ + Dox complex resulted in increased cytotoxic activity of the peritoneal macrophages of tumor-bearing mice. Cytotoxic indices of the peritoneal phagocytes in treated animals were comparable with those in the intact mice.

**Fig. 4:**
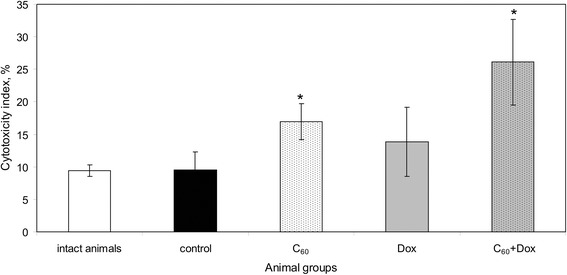
The effect of treatment with C_60_ fullerene, Dox, and C_60_ + Dox complex on the cytotoxic activity of mononuclear splenic leukocytes towards autologic tumor cells in LLC-bearing mice; *the differences are statistically valid compared to the *control* and *intact animals* (*t* test; *p* < 0.05)

Cytotoxic activity of splenic mononuclear leukocytes in tumor-bearing mice did not differ from that in the intact animals (Fig. 4).

Treatment with Dox resulted in an increase of splenocyte cytotoxicity. A significant individual variability of cytotoxic indices in animals from this group should be noted. Cytotoxicity indices of the mononuclear splenic leukocytes in animals treated with С_60_ fullerene used alone and in С_60_ + Dox complex were significantly higher than those in the untreated tumor-bearing mice. The most positive effect was observed in animals receiving С_60_ + Dox complex. Cytotoxic activity of splenic mononuclear cells towards autologic tumor cells is substantially mediated by splenic NK cells [31]. Turabekova M et al. reported that С_60_ fullerene might be recognized by Toll-like receptors (TLRs) [32]. TLR-dependent stimulatory effect of the preparation and an increased stress sensitivity of LLC cells associated with a decreased HSP70 expression might be potential reasons of increased cytotoxicity of splenic mononuclear cells in animals receiving С_60_ + Dox complex.

## Conclusions

The results of performed experiments demonstrated that treatment of mice bearing LLC with C_60_ + Dox nanocomplexes is associated with a significant antitumor effect, namely, (1) the volume of tumor of mice treated with C_60_ + Dox complex was 1.4 times smaller than that in the *control* untreated animals; (2) the number of metastatic foci in lungs of animals of the group treated with C_60_ + Dox complex was two times smaller than that in *control* untreated animals; (3) there were no metastatic foci with diameter ≥3 mm in mice treated with C_60_ + Dox complex. Western blot analysis of tumor cell lysates of animals treated with C_60_ + Dox complex revealed a significant decrease in the HSP70 level. The MTT assay showed that C_60_ + Dox complex modulates immunological reactivity of tumor-bearing mice. The potential mechanisms of C_60_ + Dox complex antitumor effect are likely to be based on its direct action on tumor cells with inducing cell death as well as an increasing of stress sensitivity and immunomodulating effect. Thus, the C_60_ + Dox nanocomplexes might be proposed as new pharmacological agents that are effectively killing tumor cells and simultaneously stimulating immune responses in tumor-bearing mice.
